# Mechanistic Insights into the Reaction of Wulfenite with Aqueous Sodium Sulfide Solution and Its Industrial Implications

**DOI:** 10.3390/molecules29225404

**Published:** 2024-11-15

**Authors:** Zi Cai, Jialei Li, Shuai Ning, Ruizeng Liu

**Affiliations:** 1 Faculty of Land Resources Engineering, Kunming University of Science and Technology, Kunming 650093, China; 17787106787@163.com (Z.C.); shuaining1996@163.com (S.N.); 2 Yunnan Key Laboratory of Green Separation and Enrichment of Strategic Mineral Resources, Kunming 650093, China; 3 School of Minerals Processing and Bioengineering, Central South University, Changsha 410083, China; 4 State Key Laboratory of Complex Nonferrous Metal Resources Clean Utilization, Kunming 650093, China

**Keywords:** wulfenite, sulfidization mechanism, sodium sulfide, interface-coupled precipitation–dissolution

## Abstract

The purpose of this study was to investigate the reaction mechanism of wulfenite with an aqueous sodium sulfide solution and thereby provide guidance for the sulfidization flotation and sodium sulfide leaching of wulfenite. For this purpose, dissolution/leaching behavior analysis, X-ray diffraction (XRD), Raman spectroscopy, X-ray photoelectron spectroscopy (XPS), and field-emission scanning electron microscopy (FESEM) were performed. The dissolution/leaching analysis indicated that sodium sulfide can induce the dissolution of PbMoO_4_. The XRD and Raman spectra results demonstrated that PbMoO_4_ was replaced by PbS at the wulfenite–sodium sulfide solution interface, and the sulfidized wulfenite particles had a PbMoO_4_/PbS core–shell structure. The XPS results also indicated the transformation of PbMoO_4_ to PbS. The FESEM images showed the growth of PbS nanoparticles on the surface of wulfenite and the dissolution pits after treatment with sodium sulfide solution. These findings showed that wulfenite sulfidization proceeds through an interface-coupled dissolution–precipitation mechanism. In the presence of sodium sulfide solution, the less stable PbMoO_4_ dissolves, and the more stable PbS phase precipitates, both of which are coupled at the wulfenite–sodium sulfide aqueous solution interface.

## 1. Introduction

Molybdenum, a rare strategic material in modern industry, has high strength, good thermal conductivity, and significant electrical conductivity even under high-temperature conditions. Due to these outstanding properties, molybdenum is widely used in many fields such as the steel industry, aerospace industry, nuclear industry, chemical industry, etc. [1,2,3,4,5]. Molybdenite (MoS_2_) is the most important raw material for molybdenum extraction [6,7]. However, with the consumption of high-quality molybdenum resources, the recovery of other molybdenum-bearing minerals such as wulfenite (PbMoO_4_) has also received increasing attention [8,9,10].

Wulfenite is generated in the weathering zones of lead–zinc sulfide deposits containing the molybdenum sulfide minerals [11]. Wulfenite deposits have been discovered in several well-known mining areas worldwide, as mentioned in the previous work [9]. According to the chemical formula of this mineral, both lead and molybdenum are available resources. Various concentration techniques have been tested for wulfenite, including flotation, leaching, gravity concentration, and the combination of beneficiation–metallurgy [12]. Song et al. [13] studied the effect of benzohydroxamic acid, sodium dodecyl sulfonate, and sodium oleate on wulfenite flotation behaviors. According to their findings, the flotation behaviors of wulfenite with these three collectors are significantly influenced by the pH of the pulp, and neutral and weakly alkaline conditions are more favorable for flotation. However, for the vast majority of non-sulfide minerals of base metals, the sulfidization flotation process usually offers higher selectivity and separation efficiency [14,15,16,17,18]. According to the previous studies [19,20], sulfidization followed by sulphydryl collector flotation can also achieve a good flotation performance for wulfenite, indicating that sodium sulfide is an effective activator for wulfenite flotation. For wulfenite leaching, sodium hydroxide and sodium sulfide are two commonly used leaching agents. When sodium hydroxide is used for leaching, both molybdenum and lead can be leached into the solution phase, requiring further Mo–Pb separation. With the use of sodium sulfide, molybdenum can leach into the leaching solution whereas lead remains entirely in the solid residue, resulting in an effective Mo–Pb separation. However, sodium sulfide leaching needs to be carried out under high-temperature conditions, resulting in high energy consumption and hydrogen sulfide pollution [9].

Notably, the processes of both sulfidization flotation and sodium sulfide leaching involve the reaction of wulfenite with an aqueous sodium sulfide solution. A rare experimental study on the sulfidization mechanism of wulfenite involved scanning electrochemical microscopy and X-ray photoelectron spectroscopy (XPS), which revealed an increase in the conductivity and the chemical changes in wulfenite surfaces after sulfidization, respectively [8]. However, there is still a data gap in terms of the changes in surface morphology and phase composition after wulfenite sulfidization, hindering a deeper understanding of the sulfidization mechanism. In this study, the phase composition was determined using X-ray diffraction (XRD) and Raman spectroscopy, the surface chemical state was investigated using XPS, and the change in surface morphology was observed via field-emission scanning electron microscopy (FESEM). The obtained results were integrated to further probe the reaction mechanism of wulfenite with an aqueous sodium sulfide solution, and the industrial implications behind this mechanism were also discussed. This study can enhance the understanding of the sulfidization of wulfenite and provide valuable guidance for the sulfidization flotation and sodium sulfide leaching of this mineral resource.

## 2. Results and Discussion

### 2.1. Dissolution/Leaching Behavior of Wulfenite

**Figure 1:**
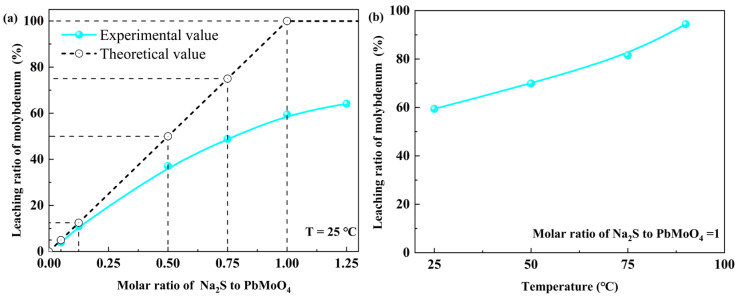
Leaching ratio of molybdenum as a function of (**a**) the molar ratio of Na_2_S to PbMoO_4_ and (**b**) temperature.

The effect of the molar ratio of Na_2_S to PbMoO_4_ and temperature on the dissolution/leaching behavior of wulfenite was investigated. As shown in Figure 1a, with the increase in the molar ratio of Na_2_S to PbMoO_4_, as shown, the molybdenum leaching ratio increased from 3.91% to 64.15%. According to the previous study [21], the reaction of wulfenite with an aqueous sodium sulfide could be written as Reaction (1). The aforementioned reaction equation demonstrates that the theoretical leaching ratio is equal to the molar ratio of Na_2_S to PbMoO_4_, provided that the molar ratio of Na_2_S to PbMoO_4_ falls within the range of 0 to 1. However, the experimental leaching ratio was lower than the theoretical value; furthermore, the gap widened as the molar ratio of Na_2_S to PbMoO_4_ approached 1. The effect of the leaching temperature is presented in Figure 1b. As the leaching temperature increased from 25 °C to 90 °C, the leaching ratio increased from 59.44% to 94.43%, indicating that high temperature is conducive to the leaching of molybdenum.

PbMoO_4_ + Na_2_S → PbS + Na_2_MoO_4_ (R1)

### 2.2. Phase Composition Determination

XRD and Raman spectroscopy were the most and second-most important methods for phase determination, respectively. In this study, these two analysis techniques were both performed to determine the phase composition of the reaction product of wulfenite with a Na_2_S aqueous solution.

**Figure 2:**
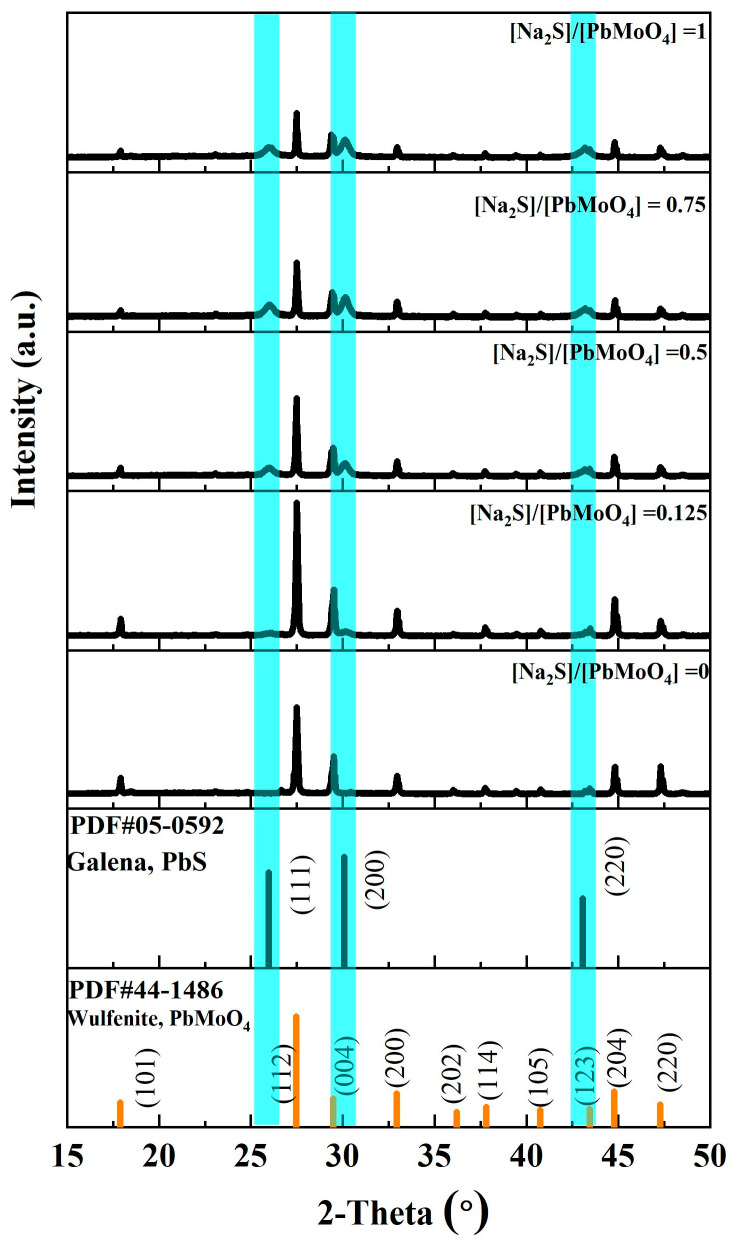
XRD patterns of wulfenite samples treated with different molar ratios of Na_2_S to PbMoO_4_.

Figure 2 shows the XRD pattern of the raw wulfenite sample, which exhibited relatively strong peaks at 17.8°, 27.6°, 29.5°, 32.9°, 35.9°, 37.8°, 40.9°, 43.6°, 45.0°, and 47.4°, corresponding to (101), (112), (004), (200), (202), (114), (105), (123), (204), and (220) of PbMoO_4_ (JCPDS No. 44-1486), respectively. These diffraction peaks corresponding to wulfenite were sharp and intense, suggesting that it was highly crystalline. In addition, no impurity peak was observed, further demonstrating the high purity of the wulfenite sample. After treatment with sodium sulfide solution, new peaks appeared at 25.9°, 30.0°, and 43.0°, which can be well indexed to PbS (JCPDS No.05-0592). These emerging peaks were broad and weak, indicating that PbS has a very small crystal size. With the increase in sodium sulfide concentration, the intensity of the peak corresponding to wulfenite was decreased, while that corresponding to PbS was increased, indicating an increase in the PbS content and a decrease in the PbMoO_4_ content.

**Figure 3:**
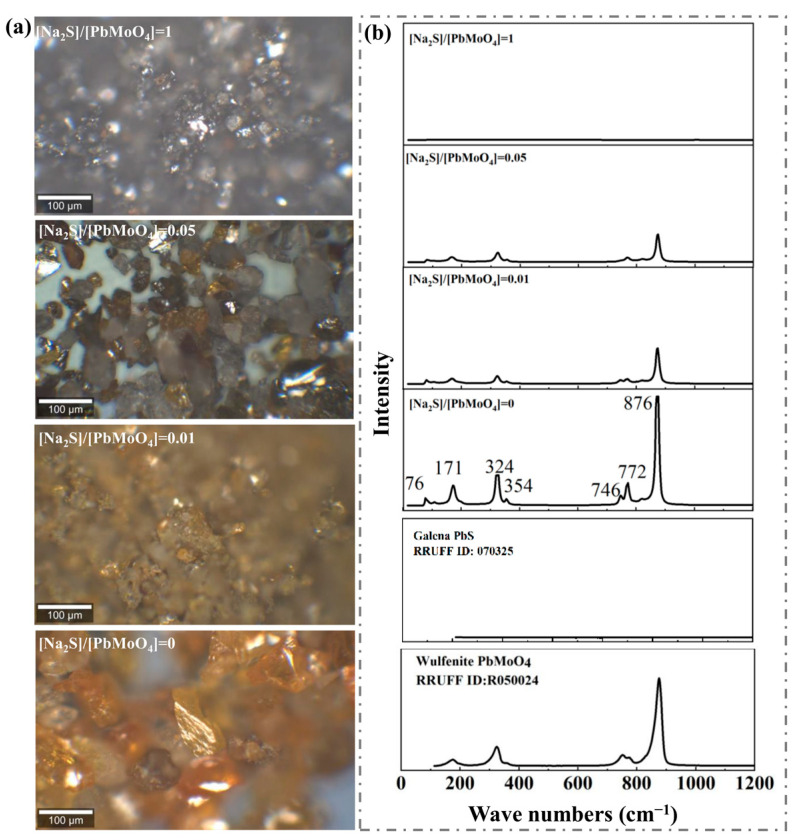
Optical micrographs (**a**) and corresponding Raman spectra (**b**) of wulfenite samples treated with different molar ratios of Na_2_S to PbMoO_4_.

Figure 3 shows the optical micrographs of the raw and sulfidized wulfenite particles and their corresponding Raman spectra. As shown in Figure 3a, light microscopy revealed that the raw wulfenite particles were yellowish-brown and semitransparent and had an adamantine luster. However, the wulfenite particles went darker with the increase in the Na_2_S/PbMoO_4_ molar ratio. In particular, with a Na_2_S/PbMoO_4_ molar ratio of 1, the optical microscopy showed that most of the imaging areas became reflective and metallic black, similar to galena, which is a mineral of PbS. Therefore, the light microscopy results provided intuitive evidence for the transformation of PbMoO_4_ to PbS.

As shown in Figure 3b, the Raman spectrum of the raw wulfenite sample exhibited seven band modes at 76, 171, 324, 354, 746, 772, and 876 cm^−1^, which are well consistent with the PbMoO_4_ data from the RRUFF Project (https://rruff.info, accessed on 15 September 2023) and the previous literature [22,23]. All of these seven Raman modes resulted from the MoO_4_ group. With the increasing Na_2_S/PbMoO_4_ molar ratio, the intensities of these seven peaks decreased; and these peaks almost disappeared at a Na_2_S/PbMoO_4_ molar ratio of 1. However, unlike the XRD patterns, no new Raman peaks emerged after sulfidization, which was due to the lack of Raman-active modes for galena PbS.

In summary, the combined findings of XRD and Raman spectroscopy clearly showed the replacement of PbMoO_4_ by PbS during wulfenite treatment with an aqueous sodium sulfide solution. Interestingly, with a Na_2_S/PbMoO_4_ molar ratio of 1, the sample exhibited strong XRD peaks, while no Raman peak was observed for PbMoO_4_. This interesting phenomenon resulted from the PbMoO_4_/PbS core–shell structure of sulfidized wulfenite particles: after sulfidization, the surface of PbMoO_4_ was replaced by PbS that is reflective and shows strong visible light absorption; therefore, the obtained light Raman signals were mostly from the surfaces of the sulfidized particles [16]. However, the XRD signals were from the entire bulk of the sulfidized particles due to the significant penetration ability of the X-rays.

### 2.3. Surface Chemical Characterization

**Figure 4:**
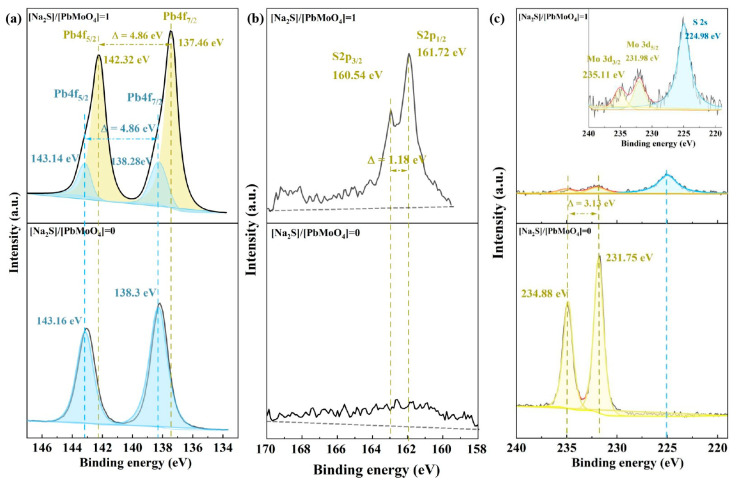
High-resolution XPS (**a**) Pb 4f, (**b**) S 2p, and (**c**) Mo 3d and S 2s spectra of raw and sulfidized wulfenite.

High-resolution XPS spectra were used to characterize the surface chemical changes before and after wulfenite sulfidization. In this regard, scans for the Pb 4f, S 2p, Mo 3d, and S 2s orbitals were recorded and depicted in Figure 4.

The XPS Pb 4f line is composed of spin–orbit doublets 4f_7/2_ and 4f_5/2_ with a peak area ratio of 4:3 and an energy separation of 4.86 eV. For the raw wulfenite surface, as shown in Figure 4a, a compliant Pb 4f_7/2_-4f_5/2_ spin–orbit doublet appeared at 143.16 eV and 138.30 eV, which resulted from PbMoO_4_ [24]. After sulfidization, the intensities of the doublets from Pb in PbMoO_4_ weakened; in addition, new Pb 4f_5/2_ and 4f_7/2_ doublets emerged at 142.32 eV and 137.46 eV, respectively, which can be well attributed to PbS [25].

Figure 4b shows the raw and sulfidized sample spectra in the binding energy range of the S 2p line. Without sulfidization, a diffuse and less sharp peak was observed throughout the range of 158 to 170 eV, which should be assigned to the energy loss peak of the Pb 4f line. The same situation has also been found in the previous studies on the sulfidization of cerussite (PbCO_3_) and anglesite (PbSO_4_) [16,26]. Therefore, attention should be given to the negative interference caused by the energy loss peak on assignment and element concentration calculations in the studies of the sulfidization of non-sulfide lead minerals. For the sulfidized wulfenite, in addition to the energy loss peaks of the Pb 4f line, a S 2p_3/2_-2p_1/2_ spin–orbit doublet with an energy separation of 1.18 eV emerged at 160.54 and 161.72 eV, matching well with the S in PbS [26].

The XPS Mo 3d line consists of spin–orbit doublets 3d_5/2_ and 3d_3/2_ with a peak area ratio of 3:2 and an energy separation of 3.13 eV. The S 2s orbital has no spin–orbital splitting and appears as a single peak in the XPS spectrum. Importantly, there is a certain overlap in the positions of these two kinds of orbital peaks. The results for the scan of the Mo 3d and S 2s orbitals for the raw and sulfidized wulfenite are shown in Figure 4c. As expected, for the raw wulfenite, spin–orbitals of Mo 3d_5/2_ and 3d_3/2_ appeared at 231.75 and 235.11 eV, respectively; and no S 2s peak was observed. After sulfidization, the intensities of the Mo 3d doublets significantly decreased; moreover, a single and symmetric peak emerged at the binding energy of 224.98 eV, which can be assigned to the S in PbS [27].

The XPS results also showed the transformation of PbMoO_4_ to PbS during wulfenite sulfidization, which is well consistent with the results of the phase composition determination.

### 2.4. Surface Morphology Observations

To better understand the sulfidization mechanism of wulfenite, FESEM was performed to observe the surface morphology of wulfenite sulfidized with different molar ratios of Na_2_S to PbMoO_4_.

**Figure 5:**
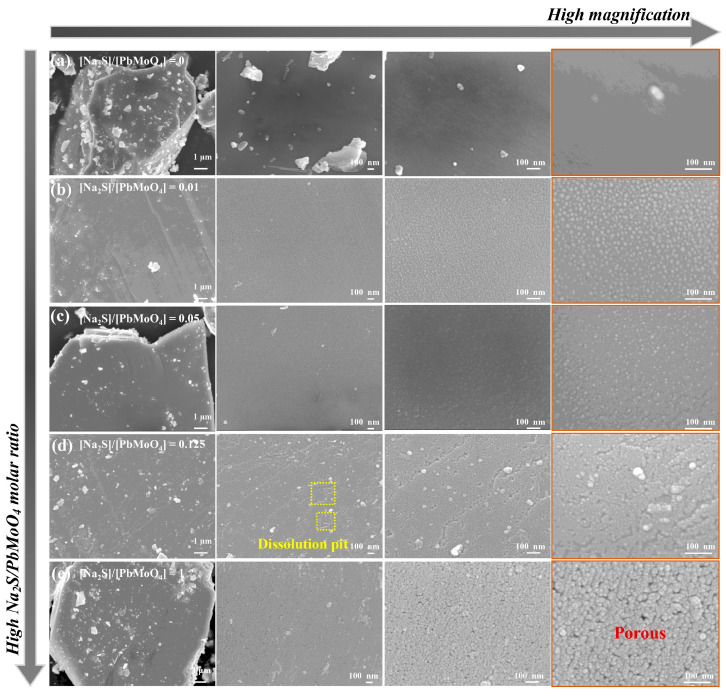
FESEM images at different magnifications of wulfenite particles sulfidized with different molar ratios of Na_2_S to PbMoO_4_.

Figure 5a shows the surface morphology of the raw wulfenite at different magnifications. As shown, due to the perfect cleavage of wulfenite, the wulfenite particles obtained by grinding exhibited a plate-like shape. Some plate-shaped protrusions of different sizes were observed on the surface of the particle; however, the image at high magnification also showed flat areas.

In contrast, sulfidization treatment resulted in significant morphological changes. As can be seen from the FESEM images at higher magnification, a greater number of nanoparticles were generated at the surfaces of the sulfidized wulfenite particles. Based on our previous findings, these nanoparticles can be regarded as PbS. The crystal size of the formed PbS products is much less than 100 nm, which is responsible for the broad and weak XRD peaks. Different Na_2_S/PbMoO_4_ molar ratios produced different morphologies of the sulfidization product layer; the condition with higher ratios appeared to produce a thicker layer of PbS. In addition, dissolution pits with nearly regular parallelogram shapes (marked in yellow in Figure 5d) were observed, revealing the dissolution of PbMoO_4_ during sulfidization. The pores of the sulfidization product layer were also observed from the high-magnification images in Figure 5e.

### 2.5. Reaction Mechanism and the Associated Industrial Implications

Sulfidization of non-sulfide minerals is essentially a phase transition process in which the surfaces of non-sulfide minerals are replaced by their corresponding sulfide components [15,16]. Recently, interface-coupled dissolution–precipitation (ICDP) has been recognized as a universal mechanism for the replacement of one solid phase by another during solid–aqueous solution interaction [28]. The ICDP process is driven by the difference in solubility between the parent phase and the product phase. This study provides evidence of the phase composition, surface chemistry, and surface morphology for the precipitation of PbS during wulfenite–sodium sulfide aqueous solution interaction. The dissolution of PbMoO_4_ may be proven by the leaching analysis of molybdenum and the presence of the dissolution pits.

PbS (*K_sp_* = 10^−27.47^) has a solubility product constant ~12 orders of magnitude smaller than PbMoO_4_ (*K_sp_* = 10^−15.62^) [15,29]. Therefore, wulfenite sulfidization can be considered to occur through the ICDP mechanism: For wulfenite–aqueous solution, dissolution equilibrium occurs as shown in Reaction (2), releasing Pb^2+^ and MoO_4_^2−^ into the solution phase and forming a fluid boundary layer due to the limited diffusion of the solute. Upon contact with a sodium sulfide solution, the fluid layer becomes oversaturated for PbS. Subsequently, PbS nanoparticles nucleate and grow on the surface of wulfenite, i.e., PbS precipitates (Reaction (3)), because the energy barrier for heterogeneous nucleation is well known to be smaller than that for homogeneous nucleation [30]. Based on Le Chatelier’s principle, the precipitation of PbS promotes the dissolution of PbMoO_4_, which in turn accelerates the generation of PbS because both lead to nonequilibrium conditions; that is, in the presence of sodium sulfide solution, the dissolution of the less stable PbMoO_4_ and the precipitation of the more stable PbS phase are coupled at the wulfenite–sodium sulfide aqueous solution interface. The total reaction of wulfenite sulfidization can be written as Reaction (4). The changes in the enthalpy and Gibbs free energy of this sulfidization reaction from 5 to 105 °C are presented in Table S1. The negative values of the enthalpy change and Gibbs free energy change demonstrated the exothermic and spontaneous nature of this reaction; that is, the reaction of wulfenite with an aqueous sodium sulfide solution is thermodynamically favorable in this temperature range.

(R2) $$\mathrm{Dissolution}:\;{\mathrm{PbMoO}}_{4}\;⇋\;{{\mathrm{Pb}}^{2+}}_{\left(\mathrm{aq}\right)}+{{{\mathrm{MoO}}_{4}}^{2-}}_{\left(\mathrm{aq}\right)}$$

(R3) $$\mathrm{Precipitation}:\;{{\mathrm{Pb}}^{2+}}_{\left(\mathrm{aq}\right)}+{S^{2-}}_{\left(\mathrm{aq}\right)}\;⇋\;{\mathrm{PbS}}_{\left(s\right)}$$

(R4) $$\mathrm{Wulfenite}\;\mathrm{sulfidization}:\;{\mathrm{PbMoO}}_{4}+{S^{2-}}_{\left(\mathrm{aq}\right)}\;\;⇋\;{\mathrm{PbS}}_{\left(s\right)}+{{{\mathrm{MoO}}_{4}}^{2-}}_{\left(\mathrm{aq}\right)}$$

Like with other non-sulfide minerals of base metals [14,15,16,17], the flotation recovery of wulfenite vs. sodium sulfide dose shows an inverted U-shaped curve [19,20]. With a moderate dose of sodium sulfide, the layer of PbS nanoparticles with adequate coverage forms on the surface of wulfenite, thereby promoting the wulfenite flotation with sulphydryl collectors. However, once the sodium sulfide dose exceeds the optimal value for flotation, the formed PbS shell will limit the further diffusion of sulfide ions into the wulfenite–solution interface. The remaining sulfide ions in the solution can lower the redox potential of the pulp and lead to competitive adsorption against sulphydryl collectors, thus depressing the flotation [31]; that is, precision management of the sodium sulfide dosage is crucial for the sulfidization flotation of non-sulfide minerals. In addition, dissolved oxygen in pulp could be depleted due to the oxidation of sodium sulfide, which is deleterious to the interaction of the formed PbS nanoparticles with sulphydryl collectors. In this regard, the sulfidization flotation performance may be improved by aerating with agitation for a while after sulfidization and before the addition of a collector.

During the experiment, the wulfenite powder turned black upon contact with the sodium sulfide solution. In addition, Figure 1 also shows that a higher reaction extent was obtained with a lower Na_2_S/PbMoO_4_ molar ratio. Therefore, wulfenite leaching using sodium sulfide is both thermodynamically and kinetically favorable in the initial stage; that is, the chemical reaction might be the rate-determining step in the initial stage. During the reaction, a PbS shell layer was formed with high coverage. The continuation of the reaction must involve (1) the diffusion of sulfide ions from the aqueous solution through the newly formed PbS shell layer into the wulfenite–solution interface, and (2) the diffusion out of the molybdate ions from the wulfenite–solution interface, also through the PbS shell layer, and into the solution. Therefore, in the middle and later stages of the reaction, diffusion might become a rate-determining step. The leaching process of wulfenite by sodium sulfide can well be described by the classical “shrinking unreacted-core model”.

For the interface-coupled PbMoO_4_ dissolution and PbS precipitation, PbS nanoparticles grow on the surface of PbMoO_4_ through three-dimensional heterogeneous nucleation rather than through layer-by-layer growth due to PbS with a cubic structure while PbMoO_4_ with a tetragonal structure [32]. Furthermore, PbS has a smaller molar volume and greater solubility than PbMoO_4_ (*V*_mol_(PbS) = 31.48 cm^3^/mol, *V*_mol_(PbMoO_4_) = 54.80 cm^3^/mol). All these stated conditions are conducive to the formation of pores [33]. Our FESEM images also clearly show that numerous pores exist in the PbS shell layer. Although the internal and external diffusion can proceed through the pores, the diffusion control due to the PbS shell leads to a slow leaching rate under ambient conditions. Thus, it is necessary to increase the leaching temperature to enhance diffusion, as shown in Figure 1b. Considering the high energy consumption and high hydrogen sulfide pollution, simultaneous grinding and leaching at low temperatures might be an effective method to eliminate the adverse effect of the formed PbS shell on leaching.

**Figure 6:**
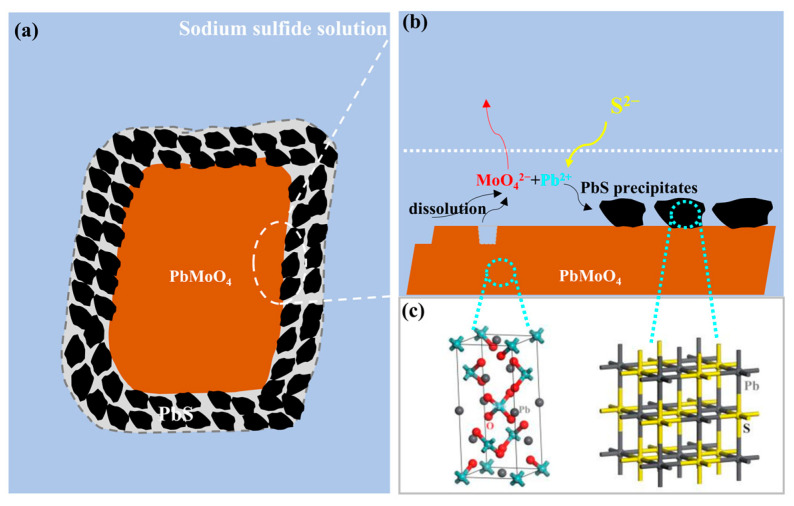
(**a**) Schematic diagram of a sulfidized wulfenite particle with a PbMoO_4_/PbS core–shell structure; (**b**) schematic diagram of wulfenite sulfidization via an ICDP mechanism; (**c**) crystal cell model of wulfenite PbMoO_4_ (Gray: Pb atom; Cyan: Mo atom; Red: O atom) and galena PbS (Gray: Pb atom; Yellow: S atom).

Based on the findings of this study, a schematic diagram of the sulfidized wulfenite particles with a PbMoO_4_/PbS core–shell structure is depicted in Figure 6a. Furthermore, the occurrence of wulfenite sulfidization via the ICDP mechanism is visually displayed in Figure 6b, and the microstructure changes before and after sulfidization can be seen in Figure 6c.

## 3. Materials and Methods

### 3.1. Materials

A high-purity natural wulfenite crystal sample with a square plate-like shape was obtained from Xinjiang, China. The bulk sample was sequentially selected by hand, ground dry, and screened to obtain the 38–72 μm size fractions for use in the study. Chemical analysis results (Table 1) showed that the obtained sample contained 25.43 wt.% Mo (based on DZG93-01.14.1) and 54.64 wt.% Pb (based on DZG20.01-2011), indicating a purity of 96.8% on Pb metal basis and a purity of 97.3% on Mo metal basis for PbMoO_4_, respectively. In addition, the sample contained 0.42 wt.% WO_3_ and 0.56 wt.% CaO. Analytical-grade sodium sulfide (Na_2_S·9H_2_O) was applied. All the experiments were conducted with high-purity water prepared with a Milli-Q IQ 7000 system (Merck, Darmstadt, Germany).

### 3.2. Sulfidization Leaching Procedure

The sulfidization treatment was performed in a thermostat water bath equipped with magnetic stirring. During each operation, 0.755 g of wulfenite powder (approximately equal to 0.002 mol PbMoO_4_) was placed in a 50 mL glass bottle. Subsequently, 20 mL of sodium sulfide solution at the given concentration was mixed with the wulfenite sample, and the beaker mouth was sealed with plastic wrap to prevent the liquid evaporating. The volume of leaching liquid was measured and then sealed in test tubes to analyze the molybdenum content.

Figure S1 shows the effect of the leaching time on the leaching ratio when the molar ratio of Na_2_S to PbMoO_4_ was kept at 1. As shown, as the leaching time increased from 10 to 30 min, the leaching ratio increased from 32.35% to 59.45%; however, when the leaching time increased to 40 min, the leaching ratio no longer increased significantly. Therefore, the 30 min leaching time was selected to investigate the effect of the molar ratio of Na_2_S to PbMoO_4_ and temperature on the dissolution/leaching behavior of wulfenite.

### 3.3. Characterizations

The pulp was stirred for 30 min at 25 °C to achieve the sulfidization of wulfenite. After sulfidization, the solid particles were separated from the leaching liquid. The solid particles were washed three times and dried under vacuum for the characterization.

XRD patterns were recorded via a Rigaku Ultima IV diffractometer (Rigaku, Akishima-shi, Japan) with Cu K-alpha-1 radiation (λ = 1.5406). Raman spectra were collected using a Raman imaging microscope (WITec Ran alpha300R, WITec, UIm, Germany) with a 532 nm line of a He–Ne laser. XPS spectra were obtained with an X-ray photoelectron spectrometer (Thermo Scientific K-Alpha, Thermo Fisher Scientific, Waltham, MA, USA) with Al Kα radiation. The obtained data were analyzed using Advantage 5.9 software and calibrated using the C1s peak at 284.8 eV corresponding to adventitious carbon. The surface morphologies of the wulfenite particles before and after sulfidization were imaged by a field-emission scanning electron microscope (JSM-7610FPlus, JEOL, Kyoto, Japan); before imaging, platinum spraying on the sample particle surface was performed to improve the surface conductivity. The molybdenum content in the leaching liquid was determined via the thiocyanate spectrophotometric method. The leaching ratio of molybdenum was calculated based on Equation (1).

(1) $$\mathrm{Leaching}\;\mathrm{ratio}=\frac{C\times V}{\omega_{s}\times m\times 1000}\times 100\%$$

where C is the molybdenum content in the leaching liquid (mg/L), *V* is the volume of the leaching liquid (0.02 L), $\omega_{s}$ is the molybdenum content of the wulfenite sample (25.4 wt.%), and *m* is the mass of the used wulfenite sample (0.755 g).

## 4. Conclusions

In this study, the reaction mechanism of wulfenite with an aqueous sodium sulfide solution was investigated using various characterization techniques. Some important and novel insights are obtained as follows:

(1) Wulfenite sulfidization proceeds through the ICDP mechanism. In the presence of sodium sulfide solution, the dissolution of the less stable PbMoO_4_ and the precipitation of the more stable PbS phase are coupled at the wulfenite–sodium sulfide aqueous solution interface.
(2) For the process of PbS precipitation, PbS nanoparticles grow on the surface of wulfenite through three-dimensional heterogeneous nucleation and growth.
(3) Sulfidized wulfenite particles have a PbMoO_4_/PbS core–shell structure. The PbS shell layer can restrict the diffusion of the reactant and the product, thereby limiting further reactions.

## Figures and Tables

**Table 1:** Chemical analysis results of the wulfenite sample.

	Pb (wt.%)	Mo (wt.%)	Purity (%) on Pb Basis.	Purity (%) on Mo Basis.
Theoretical value	56.43	26.13	100.00	100.00
Analyzed value	54.64	25.43	96.83	97.32

## Data Availability

Data are contained within the article and Supplementary Materials.

## Supplementary Materials

The following supporting information can be downloaded at: https://www.mdpi.com/article/10.3390/molecules29225404/s1, Figure S1: Effect of leaching time on the leaching ratio; Table S1: ΔH, ΔS, and ΔG for wulfenite sulfidization (PbMoO_4_ + S^2−^ = PbS + MoO_4_^2−^).
