# Supplementary material for: Mechanistic Insights into the Reaction of Wulfenite with Aqueous Sodium Sulfide Solution and Its Industrial Implications
Source: Molecules. 2024 Nov 15;29(22):5404. doi: 10.3390/molecules29225404 (PMC11596832; doi:10.3390/molecules29225404)
Supplement: Supplementary file 1 [file molecules-29-05404-s001.zip › molecules-3308380-supplementary.pdf]

Supplementary material

Mechanistic Insights into the Reaction of Wulfenite with Aqueous Sodium Sulfide Solution and Its Industrial Implications

Zi Cai [1, 2], Jialei Li[1, 3, *], Shuai Ning[1, 2], Ruizeng Liu[1, 2, 4,*]

1. Faculty of Land Resources Engineering, Kunming University of Science and Technology, Kunming 650093, China;
2. Yunnan Key Laboratory of Green Separation and Enrichment of Strategic Mineral Resources, Kunming 650093, China
3. School of Minerals Processing and Bioengineering, Central South University, Changsha 410083, China
4. State Key Laboratory of Complex Nonferrous Metal Resources Clean Utilization, Kunming 650093, China

Corresponding author:

Jialei Li, E-mail: 205601042@csu.edu.cn, lijialei1993@foxmail.com; Rui-zeng Liu, E-mail: liuruizeng@126.com, liuruizeng@kust.edu.cn

Content

Figure S1. Effect of leaching time on the leaching ratio.

Table S1. ΔH, ΔS, and ΔG for wulfenite sulfidization ($PbMoO_4$+S^{2-}=PbS+MoO_4^{2-}).

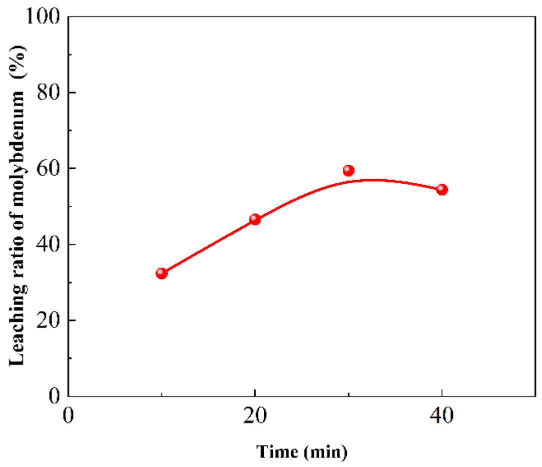

Figure S1. Effect of leaching time on the leaching ratio.

Table S1. ΔH, ΔS, and ΔG for wulfenite sulfidization reaction ($PbMoO_4+S^{2-}=PbS+MoO_4^{2-}$)

T (°C)	ΔH(kJ/mol)	ΔS(J/K/mol)	ΔG(kJ/mol)
5.000	-72.957	-19.139	-67.634
15.000	-75.365	-27.654	-67.397
25.000	-76.962	-33.107	-67.091
35.000	-78.043	-36.678	-66.741
45.000	-78.802	-39.105	-66.361
55.000	-79.346	-40.788	-65.961
65.000	-79.720	-41.912	-65.547
75.000	-80.008	-42.752	-65.124
85.000	-80.258	-43.461	-64.693
95.000	-80.512	-44.160	-64.255
105.000	-80.810	-44.957	-63.809
